# Assessing bone radiodensity and thickness in cochlear implant patients through manual photon-counting CT image segmentation using ITK-SNAP

**DOI:** 10.1038/s41598-026-45916-0

**Published:** 2026-04-24

**Authors:** Raphaële Quatre, Åsa Bonnard, Martin Eklöf, Kaijsa Edholm, Jeremy Wales

**Affiliations:** 1 Department of Oto-Rhino-Laryngology, Head and Neck Surgery, University Hospital, Grenoble Alpes, France; 2 GeodAIsics, Grenoble, France; 3 BrainTech Lab INSERM UMR 2015, Grenoble, France; 4 Medical Unit of ENT, Hearing and Balance, Karolinska University Hospital, Stockholm, Sweden; 5 CLINTEC, unit of Otorhinolaryngology, Karolinska Institutet, Stockholm, Sweden; 6 CLINTEC, unit of Radiology, Karolinska Institutet, Stockholm, Sweden

**Keywords:** Cochlear implantation, Far advanced otosclerosis, Facial nerve stimulation, Photon-counting computed tomography, Manual segmentation, Outcomes research, Translational research

## Abstract

Bone radiodensity and thickness between the cochlear implant’s middle electrodes and the facial nerve were compared across four groups—Facial nerve stimulation (FNS) patients with and without far advanced otosclerosis (FAO), and controls with and without FAO—using manual segmentation of photon counting-computed tomography (PC-CT) to assess FNS risk. This case-control study compared FAO patients with FNS (*n* = 3) to non-FAO with FNS (*n* = 2) and two control groups without FNS of post-lingually deafened patients: FAO (*n* = 2) and non-FAO (*n* = 2). Clinical data from medical record included surgical details, complications, PC-CT findings (bone thickness between the middle electrodes and the facial nerve), hearing outcomes, and implant fitting at two years. Manual segmentation of PC-CT was performed using ITK-SNAP software to measure the bone radiodensity between the mid-array electrode and the adjacent facial nerve. No significant differences were found between FNS and non-FNS patients in demographics, surgical outcomes, complications, audiometric data, or implant programming at 2 years post-surgery. A significant difference in bone radiodensity was observed in patients with FAO (1483.57 ± 122.37 HU (Hounsfield Units)) compared to those without FAO (2403.51 ± 128.24 HU; *p* < 0.001). In the non-FAO with FNS group, one patient exhibited an exceptionally short distance (0.13 mm) between the first portion of the facial nerve and the middle electrodes. When comparing FNS and non-FNS patients, no significant differences were observed in bone radiodensity (*p* = 0.85) or in bone thickness (*p* = 0.75). ITK-SNAP enables manual PC-CT segmentation to assess bone radiodensity and thickness between cochlear implant electrodes and the facial nerve. In otosclerosis, lower radiodensity alone doesn’t explain FNS, but reduced bone thickness may contribute.

## Introduction

Cochlear implantation is a standard procedure performed for patients with severe to profound sensorineural hearing loss when well-fitted hearing aids are no longer sufficient.

Facial nerve stimulation (FNS) is a rare complication of cochlear implantation, with a reported incidence ranging from 0% to 6.5%^1^. It appears to be more frequent in patients with far advanced otosclerosis (FAO), particularly in cases where straight electrodes are used (6% to 40%) rather than perimodiolar electrodes (0%)^1,2^. This complication can occur several months after cochlear implantation^3^. It is most often associated with middle electrodes located in the upper basal turn of the cochlea, near the labyrinthine segment of the facial nerve^1,3^. Several hypotheses have been discussed regarding patients with otosclerosis: reduced impedances of the otic capsule due to osteodystrophy, increased bone conductivity, and altered bone properties with the formation of bony cavities, reducing the distance between the electrode array and the intralabyrinthine portion of the facial nerve^4^. Most cases can be managed by modifying implant programming strategies: deactivating electrodes, adjusting stimulation pulse settings, or reducing stimulation intensity. However, these programming modifications may negatively impact patients’ auditory outcomes, leading to a decline in their hearing performance^1,3,4^. In rare cases, this may even necessitate cochlear implant explantation.

A high-resolution computed tomography (CT) scan and magnetic resonance imaging (MRI) are typically performed before cochlear implantation, and the quality of imaging plays a major role in anticipating potential surgical difficulties or complications^4,5^. A study by Kasetty et al. in 2019 found decreased bone thickness between the upper basal turn of the cochlea and the labyrinthine segment of the facial nerve on preoperative CT in patients with FNS after cochlear implantation^5^. Photon-counting CT (PC-CT) is an innovative imaging technology that is transforming X-ray imaging. It is based on the implementation of new photon-counting detectors, which allow the direct conversion of X-ray photons into electrical signals. This technology enhances CT image quality by improving spatial resolution, reducing signal-to-noise and contrast-to-noise ratios, and increasing spectral sensitivity while simultaneously reducing patient radiation exposure^6,7^. Several animal and cadaveric studies have demonstrated the usefulness of photon-counting technology for temporal bone imaging, showing its superiority over conventional CT that use energy-integrating detectors^7–10^.

Image segmentation is a long-established image processing technique designed to partition an image by grouping pixels into regions^11^. The main goal is to simplify image representation in space by obtaining a 3D reconstructed image allowing for a better understanding of anatomy and its variations. There are several segmentation methods: region-based segmentation, contour-based segmentation, and pixel thresholding. Image segmentation can be performed manually, semi-automatically, or automatically. Although manual segmentation is time-consuming and subject to expert interpretation, it remains the gold standard^11^. ITK-SNAP (www.itksnap.org) is a well-known interactive and open-source software for image visualization, manual and semi-automatic segmentation^12,13^. ITK is available for quantitative radiographic analysis, and some studies have demonstrated that manual segmentation using ITK-SNAP is reliable for analyzing bone density^14,15^.

To our knowledge, this is the first study to assess both bone thickness and radiodensity between the cochlear implant electrode and the facial nerve, using manual segmentation of photon-counting CT scans with ITK-SNAP.

The objective of this study was to compare bone radiodensity and thickness between the middle electrodes of the cochlear implant and the facial nerve among 4 groups: patients with FAO and FNS, patients without FAO but with FNS and two control groups without FNS: one with FAO and one without FAO. This comparison was performed using manual PC-CT image segmentation analysis with ITK-SNAP.

## Materials and methods

This was a monocentric prospective case control study conducted at a tertiary referral center between January and March 2025. Informed consent for data collection from medical records and PC-CT realization was obtained, and all procedures in the study were performed in compliance with the ethical standards of the institution and the 1964 Helsinki Declaration and its subsequent amendments. This study was approved by the Karolinska Institute, Stockholm, Sweden. IRB approval: 2023–06178-01.

All patients included in this study were over 18 years old and received a cochlear implant for severe to profound sensorineural hearing loss with a speech discrimination score below 50% for words at 65 dB SPL in silence, using a monosyllabic phonetically balanced word list, with well fitted hearing aids, according to national recommendations between 2005 and 2019^16^.

Patients with FAO and FNS were compared to a non-FAO with FNS group and to two control groups without FNS of post-lingually deafened patients, selected from the same cochlear implant database: one with FAO and one without FAO. The groups were comparable in terms of sex, age, age at implantation, period of implantation, implant type, preoperative pure tone average (mean of values at 0.5, 1, 2 and 4 kHz) and duration of hearing deprivation. The control group without FAO was composed of patients implanted for different causes of hearing loss, such as progressive hearing loss, idiopathic deafness, familial hearing loss and sudden deafness.

The collected data included age, sex, etiology, duration of hearing deprivation, age at implantation, side of implantation, implant brand, electrode type, surgical details, postoperative complications, PC-CT findings, and cochlear implant fitting outcomes at two years post-implantation. Fitting data included impedance values from both MED-EL (Innsbruck, Austria) and Cochlear systems (Sydney, Australia), and threshold (T-level) and most comfortable (C-level) levels exclusively from Cochlear as fitting data from the two brands can not be directly compared. Hearing test results at two years were also recorded.

Audiometric evaluations were performed in a soundproof room equipped with one loudspeaker positioned 1 m in front of the patient before and two years after cochlear implantation. Pure tone audiometry was performed at different frequencies (0.5, 1, 2, 4 and 8 kHz) in both ears using headphones. Free-field speech audiometry in quiet conditions was conducted at 65 dB SPL. Preoperative measurements were obtained from the ear scheduled for implantation while the patient was using a hearing aid, whereas the two-year postoperative assessments were carried out with the cochlear implant alone. Monosyllabic phonetically balanced word lists were used for these assessment^16^.

For cochlear implant programming, electrodes were divided into three groups to facilitate data interpretation: basal (electrodes 1–7 for Cochlear and 9–12 for MED-EL), middle (electrodes 8–15 for Cochlear and 5–8 for MED-EL), and apical (electrodes 16–22 for Cochlear and 1–4 for MED-EL). This classification was chosen because electrodes located in the mid-array region, corresponding to the superior portion of the basal turn, are most frequently implicated in facial nerve stimulation^1,3^.

Patients included in the study performed a PC-CT (Siemens Healthineers, Munich, Germany) scan of the temporal bone. The acquisition parameters were: 120 kV, slice thickness 0.2 mm, scanning speed 41 mm/s, pitch 0.85, 0.5s rotation time and images reconstruction using a high-resolution kernel.

Image analysis and manual region-based segmentation was performed using open-source software ITK-SNAP version 3.8.0 (Penn Image Computing and Science Laboratory, United States) to measure the mean radiodensity of the bone between the middle electrode of the cochlear implant at the upper basal turn of the cochlea and the facial nerve. A second segmentation of the implanted ear between the three portions of the facial nerve and the inner ear was also done to compare the mean radiodensity value obtained with the first segmentation.

Segmentation was performed by an Otology surgeon trained in the field of segmentation. All PC-CT images were imported as DICOM datasets and showed slices in three dimensions. Paintbrush was used for manual segmentation by drawing of the region of interest on each of the three orthogonal image slices. Intensity of voxels (Hounsfield Units (HU), number of voxels and volume (mm^3^) of the area segmented was then determined by the software and compared to the non-segmented area. Radiodensity of the segmented bone was defined as the mean radio-intensity of the segmented bone compared to the mean radio-intensity of the non-segmented area.

The thickness was defined as the shortest distance between the surface of the closest cochlear implant electrode and the facial nerve assessed independently by a senior Head and Neck radiologist and an otology surgeon on coronal view of PC-CT images using ITK-SNAP software. The average of the two evaluations was used for the results. In all patients, the shortest distance was found between the middle electrodes and either the initial segment of the facial nerve or the geniculate ganglion. Measurements were performed in the coronal plane, as this orientation provides reproducible visualization of the facial nerve in relation to the basal turn of the cochlea, enabling accurate segmentation and thickness assessment using ITK-SNAP.

Statistical analyses were performed using JASP software (V0.19.1 Intel). This study was designed as a prospective exploratory pilot study. Given the rarity of facial nerve stimulation in cochlear implant recipients with far-advanced otosclerosis and the limited availability of photon-counting CT imaging, no formal a priori power calculation was performed.

The Shapiro-Wilk test was used to assess the normality of the distribution of quantitative demographic and audiometric data. As the number of patients included in each of the four groups was small, we did not perform statistical tests comparing the four groups. To compare quantitative data between FNS and non-FNS patients or FAO and non-FAO patients the Student’s t-test was applied if the distribution was normal; otherwise, a non-parametric test such as the Mann-Whitney U-test was used. For qualitative data, the Chi-squared test was performed, while Fisher’s exact test was employed for data with fewer data points.

All quantitative measurements are expressed as means ± standard deviation (SD).

A p-value of less than 0.05 was considered indicative of statistical significance.

## Results

### Demographic and audiometric data

A total of 9 patients were included in this study, comprising 8 males and 1 female. The mean age was 66.11 ± 11.96 years, with a mean age at cochlear implantation of 56.17 ± 9.82 years. The cohort included 3 patients with FNS associated with FAO, 2 non-FAO patients with FNS, and 4 control patients without FNS. Among the controls, 2 had FAO and 2 had other etiologies (one due to ototoxicity and one of unknown origin). Seven patients were implanted on the right side, and 2 on the left. Regarding implant brands, 7 patients received Cochlear implants, while 2 received MED-EL implants.

Among patients with otosclerosis, 2 patients with FNS and 1 control had undergone prior stapedotomy.

In the FAO with FNS group, 1 straight (CI422) and 2 perimodiolar electrodes (CI512, CI24RE) were used. In the non-FAO with FNS group, 1 straight (Flex EAS) and 1 perimodiolar electrode (CI532) were used. In the control group, 3 straight (CI522 and Flex soft) and 2 perimodiolar electrodes (CI512) were used. No statistically significant difference in electrode type was observed between FAO and non-FAO patients (*p* = 0.89) or between FNS and non-FNS patients (*p* = 0.89).

For 2 patients, medical records confirmed that electrode 11 (from Cochlear) was the one associated with FNS.

Main surgical findings, complications, and mean pre- and post-operative speech comprehension scores for each group are summarized in Table 1.

**Table 1:** Main demographic, surgical, complication and audiometric data. FNS: facial nerve stimulation. FAO: Far advanced otosclerosis.

		FNS + FAO (*n* = 3)	FNS Ø FAO (*n* = 2)	CONTROLS + FAO (*n* = 2)	CONTROLS Ø FAO (*n* = 2)
Age (mean years)		66.67	64.5	64.5	68.5
Age at implantation (mean years)		56	54.21	53.88	60.67
Sex	Male	3 (100%)	1 (50%)	2 (100%)	2 (100%)
Implant brand	Cochlear^®^	3 (100%)	1 (50%)	1 (50%)	2 (100%)
Side of implantation	Right	2 (66.67%)	2 (100%)	2 (100%)	1 (50%)
Surgical approach	Round Window	3 (100%)	2 (100%)	1(50%)	2 (100%)
Scala insertion	Scala tympani	3 (100%)	2 (100%)	1(50%)	2 (100%)
Cochlear ossification	No	3 (100%)	2 (100%)	2 (100%)	2 (100%)
Full insertion	Yes	3 (100%)	2 (100%)	2 (100%)	2 (100%)
Tinnitus	Yes	1 (33.33%)	0 (0%)	2 (100%)	0 (0%)
Dizziness	Yes	3 (100%)	2 (100%)	0 (0%)	0 (0%)
Facial nerve stimulation	Yes	3 (100%)	2 (100%)	0 (0%)	0 (0%)
Speech comprehension before surgery at 65 dB SPL with hearing aids (mean ± SD)		10.67% ±13.61	16% ± 22.63	11% ± 15.56	12% ± 16.97
Speech comprehension at 2 years at 65 dB SPL with cochlear implant alone (mean ± SD)		53.33% ± 26.1	69% ± 9.9	55% ± 18.39	66% ± 8.49

No statistically significant differences were observed between FNS and non-FNS patients in terms of surgical parameters (surgical approach: *p* = 0.24; scala insertion: *p* = 0.24), complications (tinnitus: *p* = 0.34; dizziness: *p* = 0.76), audiometric data before surgery (*p* = 0.90), and at 2 years post-surgery (*p* = 0.94).

Similarly, no statistically significant differences were found between FAO and non-FAO patients regarding surgical findings (surgical approach: *p* = 0.34; scala insertion: *p* = 0.34), complications (tinnitus: *p* = 0.06; dizziness: *p* = 0.29), audiometric data before surgery (*p* = 0.75), and at 2 years post-surgery (*p* = 0.26).

### Cochlear implant programming

When comparing patients with and without FNS, there was no significant difference in impedance values at the basal (*p* = 0.87), middle (*p* = 0.88), apical (*p* = 0.32), or all electrode levels together (*p* = 0.72). Likewise, no significant impedance differences were found between FAO and non-FAO patients at the basal (*p* = 0.52), middle (*p* = 0.95), apical (*p* = 0.37), or overall electrode measurements (*p* = 0.95) (Table 2).

There were no statistically significant difference in C- or T- levels of basal, middle or apical electrodes between FNS patients and non-FNS patients (p _T−level 1−7_ =0.61; p _C−level 1−7_ = 0.69; p _T−level 8−15_ = 0.6; p _C−level 8−15_ = 0.79; p _T−level 16−22_ = 0.61; p _C−level 16−22_ = 0.69; p _T−level 1−22_ = 0.87; p _C−level 1−22_ = 0.9) (Table 3).

Moreover, there were no statistically significant difference in C- or T- levels of basal, middle or apical electrodes between otosclerosis patients and non-otosclerosis patients (p _T−level 1−7_ =0.74; p _C−level 1−7_ = 0.26; p _T−level 8−15_ = 0.52; p _C−level 8−15_ = 1; p _T−level 16−22_ = 0.39; p _C−level 16−22_ = 0.16; p _T−level 1−22_ = 0.8; p _C−level 1−22_ = 0.63) (Table 3).

**Table 2:** Impedances at two years of basal, middle, apical and total electrodes of the different groups expressed in mean ± standard deviation. FNS: facial nerve stimulation. FAO: Far advanced otosclerosis.

Time	Impedances	FNS + FAO (*n = 3*)	FNS Ø FAO (*n = 2*)	FNS TOTAL (*n = 5*)	CONTROLS + FAO (*n = 2*)	CONTROLS Ø FAO (*n = 2*)	CONTROLS TOTAL (*n = 4*)	FAO (*n = 5*)	Ø FAO (*n = 4*)
		Mean ± SD (kOhm)							
2 years	Basal electrodes	11.17 ± 2.84	8.83 ± 0.14	10.23 ± 2.39	10.41 ± 1.02	10.64 ± 4.64	10.53 ± 2.75	10.86 ± 2.12	9.73 ± 2.88
	Middle electrodes	9.36 ± 2.33	8.07 ± 0.05	8.84 ± 1.79	8.22 ± 0.97	9.91 ± 4.01	9.07 ± 2.57	8.9 ± 1.83	8.99 ± 2.55
	Apical Electrodes	8.49 ± 1.85	8.54 ± 0.74	8.51 ± 1.36	8.62 ± 0.02	10.07 ± 0.11	9.35 ± 0.84	8.54 ± 1.31	9.3 ± 0.98
	Total Electrodes	9.66 ± 2.1	8.45 ± 0.31	9.18 ± 1.63	9.03 ± 0.69	10.2 ± 2.97	9.61 ± 1.88	9.41 ± 1.56	9.32 ± 1.99

**Table 3:** C- and T-levels at two years, from Cochlear^®^ brand implants, of basal, middle, apical and total electrodes of the different groups expressed in mean ± standard deviation. FNS: facial nerve stimulation. FAO: Far advanced otosclerosis.

Time	Electrodes setting	FNS + FAO (*n* = 3)	FNS Ø FAO (*n* = 1)	FNS TOTAL *n* = 4	CONTROLS + FAO (*n* = 1)	CONTROLS Ø FAO (*n* = 2)	CONTROLS TOTAL (*n* = 3)	FAO (*n* = 4)	Ø FAO (*n* = 3)
		Mean ± SD (Current Level)							
2 years	T-level 1–7	129.1 ± 25.12	123	127.5 ± 20.73	118.8	121.7 ± 4.6	120.7 ± 3.677	126.5 ± 21.14	122.1 ± 3.37
	C-level 1–7	176.7 ± 15.70	169	174.8 ± 13.38	179.8	166.7 ± 4.4	171.1 ± 8.18	177.5 ± 12.91	167.5 ± 3.38
	T-level 8–15	117.9 ± 8	135.9	122.4 ± 11.13	134.3	122.9 ± 7.13	126.7 ± 8.287	122.0 ± 10.5	127.2 ± 9.03
	C-level 8–15	183.4 ± 16.09	188	184.5 ± 13.34	199.6	181 ± 1.52^6^	187.2 ± 10.82	187.4 ± 15.45	183.3 ± 4.21
	T-level 16–22	120.3 ± 15.69	112.8	118.4 ± 13.35	136.7	117.2 ± 3.97	123.7 ± 11.61	124.4 ± 15.21	115.7 ± 3.8
	C-level 16–22	179.7 ± 14.52	159.3	174.6 ± 15.64	192.3	172.7 ± 4.82	179.2 ± 11.84	182.9 ± 13.43	168.2 ± 8.5
	T-level 1–22	121.9 ± 14.34	124.4	122.6 ± 11.77	130.1	120.7 ± 5.33	123.8 ± 6.62	124 ± 12.4	122 ± 4.35
	C-level 1–22	180.2 ± 15.23	172.8	178.3 ± 12.97	191	173.8 ± 2.38	179.5 ± 10.07	182.9 ± 13.56	173.5 ± 1.78

### Bone segmentation and facial nerve proximity analysis

**Fig. 1:**
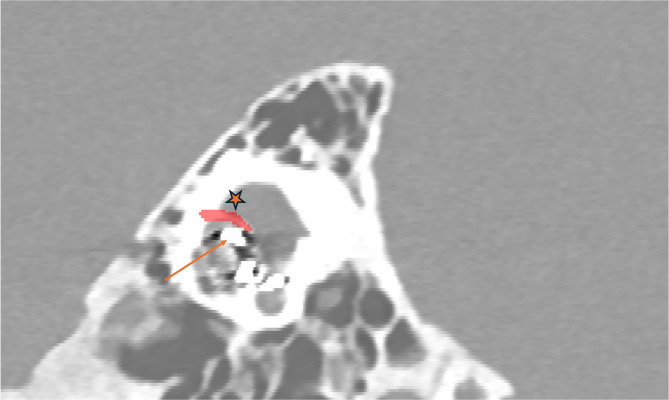
Coronal PC-CT image of the temporal bone in a non-FAO patient without FNS, showing manual bone segmentation (in red) between the middle electrode of the cochlear implant and the facial nerve using ITK-SNAP. The orange arrow indicates the cochlear implant’s middle electrodes, and the orange star marks the facial nerve.

**Fig. 2:**
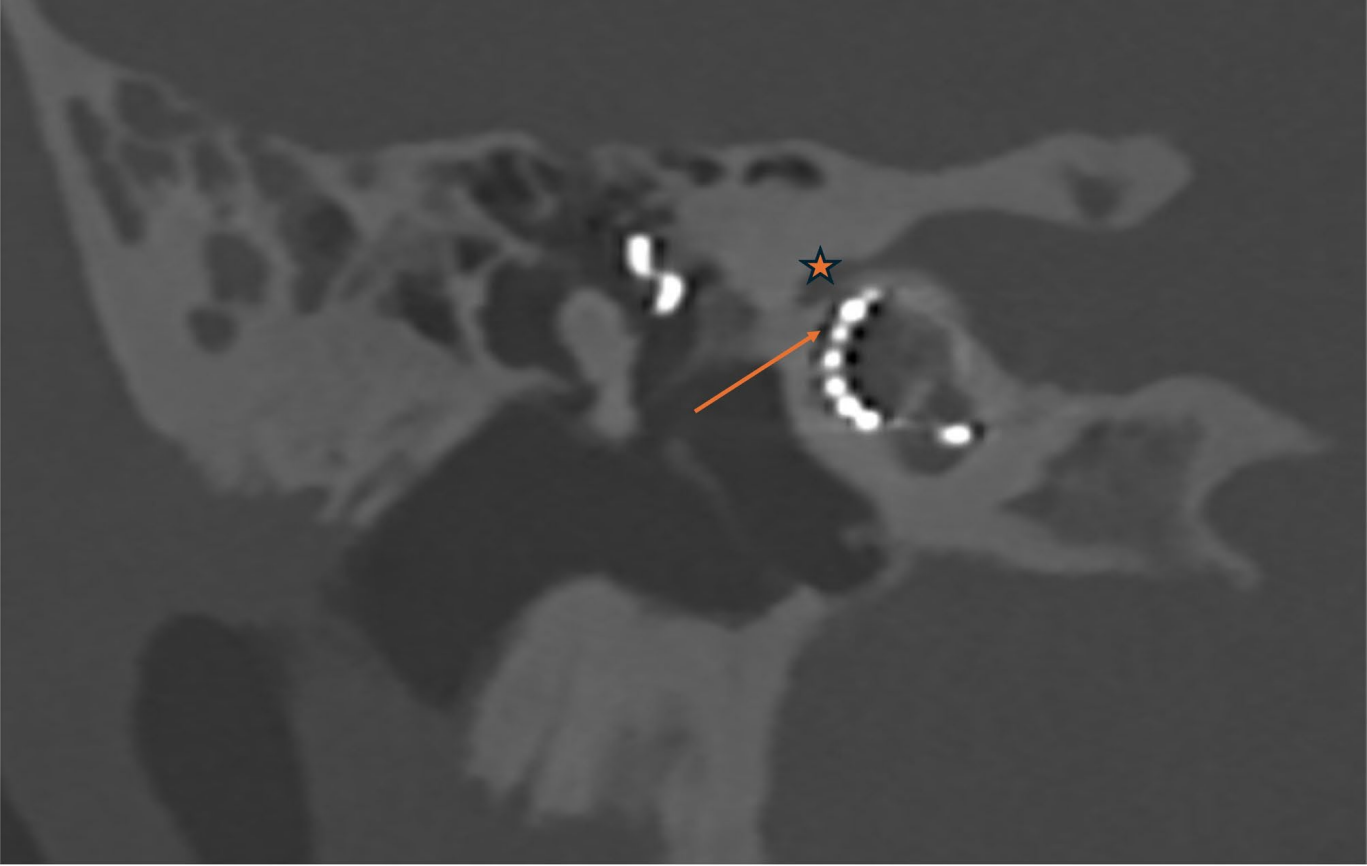
Coronal PC-CT image of the temporal bone in a non-FAO patient with FNS illustrating the proximity between the surface of the cochlear implant’s middle electrode and the facial nerve. The orange arrow indicates the cochlear implant’s middle electrodes, and the orange star marks the facial nerve.

The mean number of segmented voxels was 15,185 ± 3863, with a corresponding mean segmented volume of 18.2 ± 5.98 mm³. No statistically significant difference was found between FNS and non-FNS patients or FAO and non-FAO patients regarding the number of voxels (*p* = 0.36 and *p* = 0.69, respectively) or the segmented volume (*p* = 0.38 and *p* = 0.96, respectively). The method of segmentation is presented in Fig. 1. In the FNS group without FAO, one patient exhibited an exceptionally short distance of 0.13 mm between the first portion of the facial nerve and the middle electrodes of the cochlear implant (Fig. 2). When comparing FNS and non-FNS patients, no significant differences were observed in bone radiodensity (*p* = 0.85) or in the distance to the facial nerve (*p* = 0.75) (Table 4).

**Table 4:** Bone radiodensity (HU) of the segmented aera and the shortest distance (mm) between the middle electrodes of the cochlear implant and the facial nerve across facial nerve stimulation (FNS) patients versus non-FNS patients expressed in mean ± standard deviation.

	FNS (*n = 5*)	CONTROLS (*n = 4*)	*p*-value
	Mean ± SD		
Bone radiodensity of the segmented aera (HU)	1861.8 ± 528.99	1930.72 ± 535.41	0.85
Distance (mm)	0.58 ± 0.34	0.51 ± 0.33	0.75

**Fig. 3:**
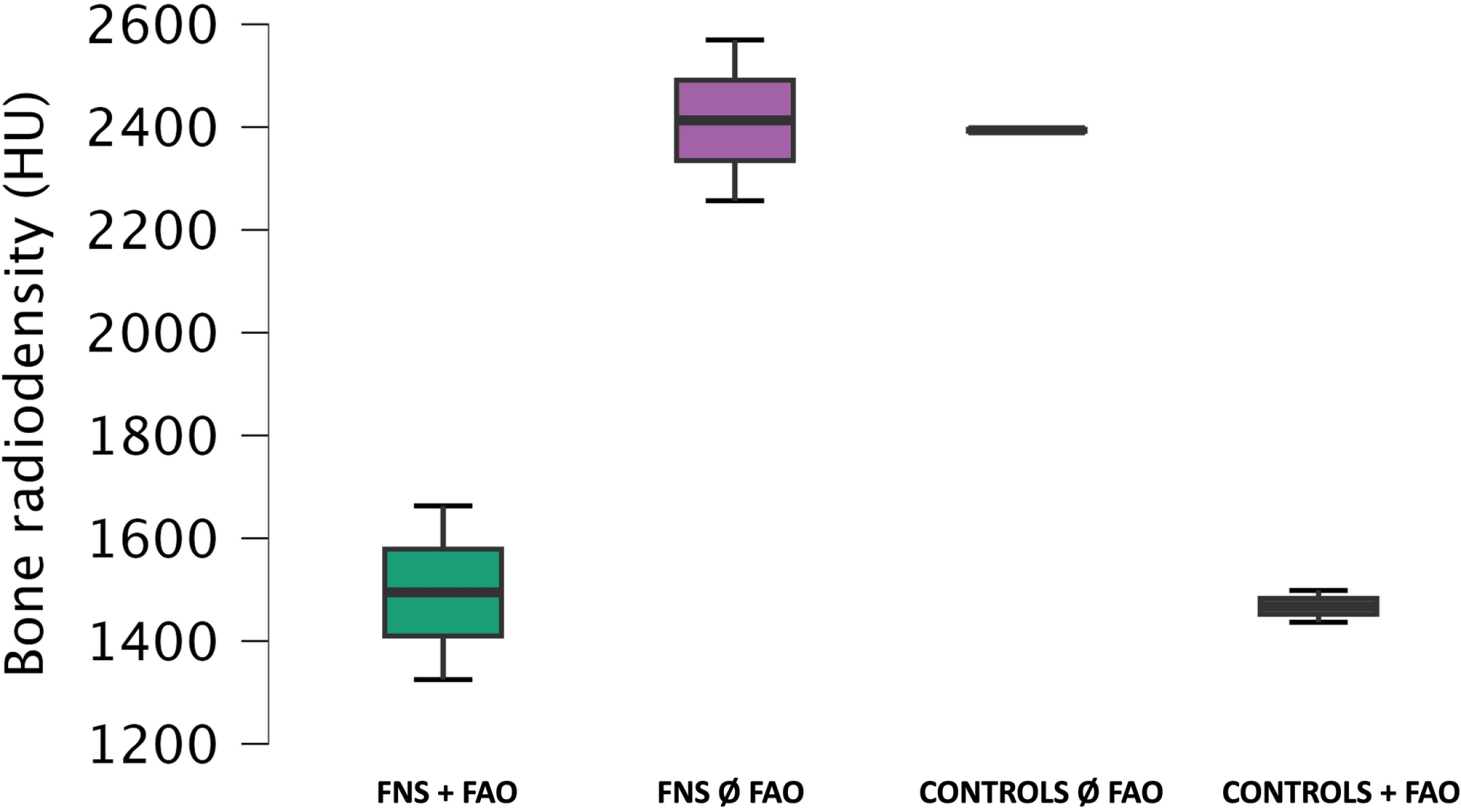
Bone radiodensity (HU) of the segmented aera across facial nerve stimulation (FNS) patients with or without far advanced otosclerosis (FAO) and control groups with and without FAO patients expressed in mean ± standard deviation.

However, a statistically significant difference in bone radiodensity was observed based on etiology. Patients with FAO had a significantly lower mean bone radiodensity (1483.57 ± 122.37 HU) compared to those without FAO (2403.51 ± 128.24 HU; *p* < 0.001) (Fig. 3). In contrast, the distance between the facial nerve and the middle electrodes did not significantly differ by etiology, with a mean of 0.62 ± 0.33 mm in FAO patients versus 0.46 ± 0.32 mm in other patients (*p* = 0.5).

## Discussion

To our knowledge, this was the first study to use PC-CT combined with manual segmentation to assess the bone radiodensity of the temporal bone. We also measured the distance between the first portion of the facial nerve and the middle electrodes of the cochlear implant.

PC-CT is a new CT imaging technique. It enhances image quality by improving spatial resolution, increasing spectral sensitivity, and reducing both the signal-to-noise and contrast-to-noise ratios. Additionally, it offers the benefit of a lower radiation dose compared to conventional CT^7,8^. These characteristics make PC-CT especially well suited for temporal bone imaging, enabling precise evaluation of fine bony details and electrode positioning that are critical for understanding mechanisms of facial nerve stimulation.

Traditionally, bone density is measured using Dual-energy X-ray Absorptiometry (DEXA)^17,18^. DEXA is considered the gold standard and was primarily designed to assess bone mineral density (BMD) in large, anatomically simple regions such as the spine, hip, and forearm. Its application to small or anatomically complex structures, like the temporal bone, is limited due to several factors. First, DEXA provides areal BMD measurements (g/cm²), which combine both cortical and trabecular bone components and may be influenced by overlying tissues and structural artifacts. Second, the spatial resolution of DEXA is insufficient to accurately delineate the fine structures of the temporal bone or cochlea, leading to potential inaccuracies in density measurements^19^.

Software tools like ITK-SNAP now allow similar measurements from CT images. ITK-SNAP is reliable and widely used to obtain localized measurements of bone thickness or density in specific regions of interest. It allows both manual and semi-automatic segmentation of regions of interest^12,13^. Manual segmentation remains the gold standard, and in our study manual segmentation was chosen due to the unusual location of the region of interest^11^. The area between the middle electrodes of the cochlear implant in the cochlea and the facial nerve was therefore segmented manually twice.

Patients with FAO showed lower bone radiodensity than non-FAO patients. This supports the validity of using PC-CT with ITK-SNAP to assess bone radiodensity. However, we did not find any statistically significant difference in bone radiodensity between FAO patients with and without FNS. Similarly, no significant difference was found between non-FAO patients with and without FNS.

One non-FAO patient with FNS had a markable short distance—only 0.13 mm—between the facial nerve and the electrode. This could explain the occurrence of FNS in that case. For other FNS cases, no clear variable could explain the FNS based on PC-CT segmentation.

The electrode responsible for facial nerve stimulation could be clearly identified in only two patients (electrode 11 in a Cochlear^®^ device), corresponding to the middle electrode region. Because the specific electrode contact responsible for facial nerve stimulation could not be consistently identified in all patients, the implant programming analysis was performed using grouped electrode regions rather than electrode-specific comparisons. This methodological choice allowed us to evaluate stimulation characteristics in the electrode region most frequently associated with facial nerve stimulation.

In our cohort, electrode impedances tended to be lower in FNS patients than in non-FNS patients. Conversely, FAO patients showed higher impedances compared to non-FAO patients, particularly in apical and total electrodes. However, these differences were not statistically significant.

Impedances were notably reduced in FNS patients without FAO. There was also a trend toward lower stimulation thresholds in all FNS patients, though it was not significant. On the other hand, FAO patients had higher stimulation levels than non-FAO patients. This finding is consistent with previous studies^20–23^.

These results may indicate that otosclerosis causes increased impedances and higher stimulation thresholds. This combination could elevate the risk of FNS, especially in the medial electrodes of the implant^24^. However, FNS patients showed an overall trend of lower stimulation thresholds. This might reflect adjustments made during programming to reduce the clinical impact of FNS on patient quality of life.

In summary, patients with both FAO and FNS tended to show lower radiodensity and slightly higher stimulation thresholds, which may be associated with an increased risk of FNS. Conversely, in FNS cases without FAO, a shorter distance to the facial nerve may predispose to FNS.

In clinical practice, facial nerve stimulation was managed through standard fitting adjustments, including reduction of stimulation levels and, when necessary, deactivation of the implicated electrode contacts.

The main limitation of this study is the small number of included patients. FNS is a rare complication of cochlear implantation. In our experience, it occurred in only 7.02% of cases, most of whom had otosclerosis^25^. Because of this low prevalence, the number of FNS cases available for comparison was limited. No formal a priori power calculation was performed, and therefore the statistical power of the study is likely limited. Consequently, the absence of statistically significant differences between the FNS and non-FNS groups should be interpreted with caution, as it may reflect insufficient power to detect true differences rather than true equivalence between groups. Additionally, heterogeneity in cochlear implant brands and electrode types may further reduce the ability to detect significant associations.

Despite this, the study demonstrates the originality and feasibility of using PC-CT with ITK-SNAP to measure temporal bone radiodensity. It clearly shows that radiodensity around the facial nerve is lower in patients with otosclerosis. This study should be considered as a feasibility investigation intended to support and inform larger, multicenter studies in the future.

## Conclusion

In this study, we demonstrated that manual segmentation of PC-CT images using ITK-SNAP is a practical method for assessing bone radiodensity and thickness in the temporal bone, specifically between the middle electrodes of the cochlear implant and the facial nerve. We confirmed that patients with otosclerosis have lower bone radiodensity than those without otosclerosis, although this difference alone does not fully account for the occurrence of FNS. Reduced bone thickness may be responsible for some cases of FNS. Further studies with larger patient cohorts and detailed electrode-specific electrophysiological data will be necessary to confirm these preliminary observations.

## Data Availability

All data generated or analysed during this study are included in this published article.
